# The Complete Sequence of a Human Parainfluenzavirus 4 Genome

**DOI:** 10.3390/v1010026

**Published:** 2009-06-02

**Authors:** Carmen Yea, Rose Cheung, Carol Collins, Dena Adachi, John Nishikawa, Raymond Tellier

**Affiliations:** 1 Program in Genetics and Genome Biology, Research Institute, Hospital for Sick Children, Toronto Ontario, Canada; E-mails: carmen.yea@sickkids.ca (C.Y.); 2 Division of Microbiology, Hospital for Sick Children, Toronto, Ontario, Canada; E-mails: carol.collins@sickkids.ca (C.C.) dena.adachi@sickkids.ca (D.A.), john.nishikawa@sickkids.ca (J.N.); 3 Department of Laboratory Medicine and Pathobiology, University of Toronto, Toronto, Ontario, Canada; 4 Current address: Provincial Laboratory for Public Health (Microbiology), Alberta, and University of Calgary, Calgary, Alberta, Canada

**Keywords:** human parainfluenza virus 4, complete genome sequence, L protein, long RT-PCR

## Abstract

Although the human parainfluenza virus 4 (HPIV4) has been known for a long time, its genome, alone among the human paramyxoviruses, has not been completely sequenced to date. In this study we obtained the first complete genomic sequence of HPIV4 from a clinical isolate named SKPIV4 obtained at the Hospital for Sick Children in Toronto (Ontario, Canada). The coding regions for the N, P/V, M, F and HN proteins show very high identities (95% to 97%) with previously available partial sequences for HPIV4B. The sequence for the L protein and the non-coding regions represent new information. A surprising feature of the genome is its length, more than 17 kb, making it the longest genome within the genus *Rubulavirus*, although the length is well within the known range of 15 kb to 19 kb for the subfamily *Paramyxovirinae*. The availability of a complete genomic sequence will facilitate investigations on a respiratory virus that is still not completely characterized.

## 1. Introduction

Human parainfluenza viruses are enveloped, negative strand RNA viruses belonging to the family *Paramyxoviridae*, and which cause respiratory tract infections. The two species human parainfluenza 1 (HPIV1) and human parainfluenza 3 (HPIV3) belong to the genus *Respirovirus*, whereas HPIV2 and HPIV4 belong to the genus *Rubulavirus*. Among the known human paramyxoviruses, the genome of HPIV4 has not yet been completely sequenced. The species HPIV4 is further divided into types HPIV4A and HPIV4B, based on antigenic differences demonstrated by hemadsorption inhibition and monoclonal antibody reactivity [1].

In this study the first complete genomic sequence of a HPIV4 was determined. It was based on a clinical isolate, designated SKPIV4, that was shown to be a HPIV4B by direct immunofluorescence microscopy and sequencing of the nucleocapsid (N) gene. The availability of this first complete sequence of HPIV4 fills an important gap in our knowledge of the *Paramyxoviridae* family and contributes to a complete description of the human virome.

## 2. Results

### 2.1. Identification of the respiratory virus isolate SKPIV4 as a HPIV4

**Figure 1.**
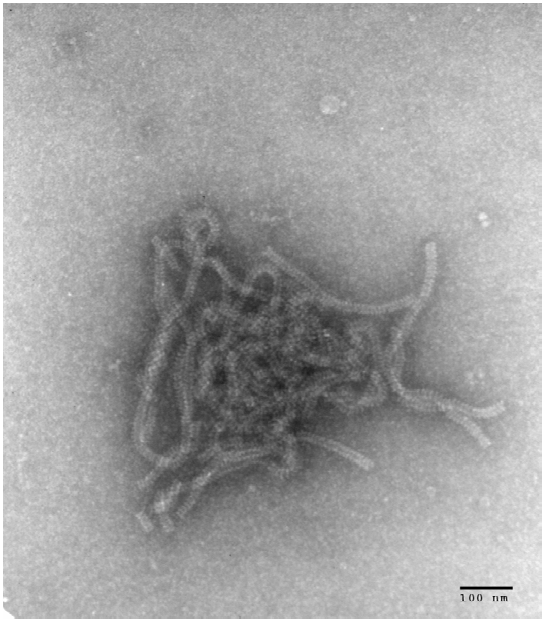
Electron microscopy photograph obtained from LLC-MK2 cells infected with SKPIV4, showing typical nucleocapsids of paramyxoviruses.

**Figure 2.**
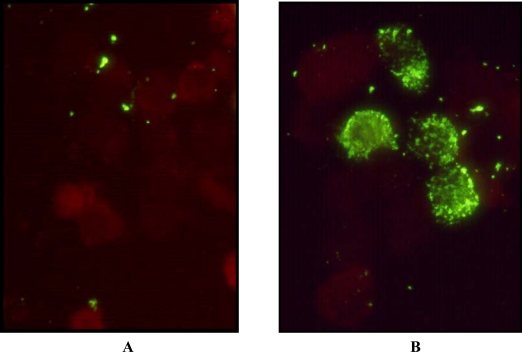
Immunofluorescence microscopy after permeabilisation, fixation and staining with FITC labeled anti HPIV4 Mab 5034 (Chemicon). Panel A, uninfected LLC-MK2 cells; Panel B, cells infected with the isolate SKPIV4.

The isolate of HPIV4 was recovered from the nasopharyngeal swab taken from a patient at the Hospital for Sick Children. The presence of a growing virus was first inferred from a positive hemadsorption reaction. Immunofluorescence microscopy on a cell pellet from the culture was negative using monoclonal antibodies (Mabs) against Influenza A and B and against HPIV1, HPIV2, and HPIV3. Electron microscopy examination of a cell pellet revealed the presence of characteristic nucleocapsids from paramyxoviruses (Figure 1). Immunofluorescence microscopy with an anti-HPIV4 Mab revealed the expected intracytoplasmic staining of cells infected with HPIV4 (Figure 2).

### 2.2. Amplification and sequencing of the viral genome

**Figure 3.**
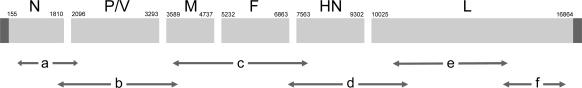
Schematic illustration of the HIPV4 genome and the relative positions of the amplicons obtained by long RT-PCR that were used in sequencing the SKPIV4 genome.

Primers to amplify large overlapping amplicons spanning most of the viral genome (Figure 3) were designed based on conserved regions in the sequence of paramyxoviruses, or from the existing partial sequence data available for HPIV4 in GenBank. The sizes of the amplicons are given in Table 1. The sequences of the genomic termini were determined by RNA ligase circularization of the genome followed by RT-PCR of an amplicon bracketing the junction, and by 5′ RACE. Additional experiments described in section 3.11 further confirmed the sequence. After assembly and editing, the complete sequence of SKPIV4 had a length of 17361 nts, and was deposited in GenBank under the accession number EU627591.

### 2.3. The Nucleocapsid (N) gene

**Figure 4.**
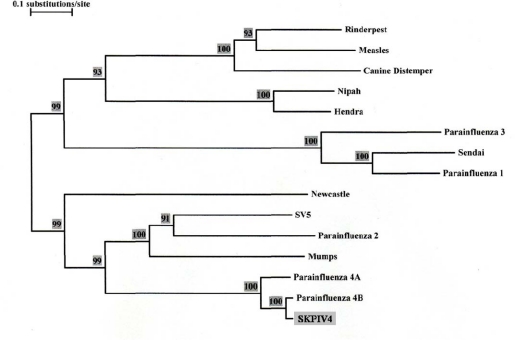
Phylogenetic tree built from an alignment of the nucleocapsid (N) ORF nucleotide sequence of several paramyxoviruses. The numbers at the nodes indicate the results of the Bootstrap analysis, expressed as percentages. The N ORF sequences were excerpted from the complete genomic sequence for Rinderpest virus (GenBank accession number NC_006296), Measles virus (AY486083), Canine Distemper virus (AY649446), Nipah virus (AY988601), Hendra virus (NC_001906), Human Parainfluenza virus 3 (EU424062), Sendai Virus (NC_001552), Human Parainfluenza virus 1 (NC_003461), Newcastle Disease virus (DQ486859), SV5 (NC_006430), Human Parainfluenza virus 2 (NC_003443), Mumps virus (AF314558). The GenBank accession numbers for the N gene sequences of Human Parainfluenza 4A and 4B are M32982 and M32983, respectively.

The first coding region in the genome contains a single ORF (155 to 1,810) encoding for the nucleocapsid. BLAST analysis showed a 97% identity to a previously determined sequence for the nucleocapsid gene of a HPIV4B (89% with HPIV4A), with a 98% identity at the amino acid (a.a) level (92% with HPIV4A) [2]. Figure 4 shows a phylogenetic tree calculated from an alignment of the sequences of the nucleocapsid ORF from several paramyxoviruses, including previously determined sequences from HPIV4A and HPIV4B isolates. Figure 4 shows conclusively that the SKPIV4 isolate should be classified as a HPIV4B.

### 2.4. The Phosphoprotein/V-protein (P/V) gene

The next coding region, P/V, from nts 2,096 to 3,293, contains potentially two ORFs through the addition of non-templated G residues to the mRNA [3]. Overall, BLAST analyses revealed a 96% identity with the corresponding coding region of a HPIV4B isolate (87% with HPIV4A), with a 100% identity within the region where insertion of non-templated Gs occur during mRNA synthesis [3]. Based on the postulated translation of these proteins, BLAST analysis revealed a 93% identity of the P protein at the a.a level between SKPIV4 and that of Kondo et al (84% with HPIV4A), and a 92% identity at the a.a. level for the V protein (81% with HPIV4A) [3].

### 2.5. The Matrix (M) gene

The ORF for the matrix (M) protein goes from nts 3,589 to 4,737. By BLAST analyses it has a 96% identity with the previously reported M gene for HPIV4B (89% with HPIV4A)[4]. At the a.a. level, an identity of 97% is observed (95% with HPIV4A).

### 2.6. The Fusion (F) gene

An ORF for the F coding gene was found to extend from nts 5,232 to 6,863. By BLAST analyses the corresponding sequence has a 96% homology to the sequence of HPIV4B previously published (90% with HPIV4A) [5], with an identity of 97% at the a.a. level (94% with HPIV4A).

### 2.7. The Haemagglutinin-Neuraminidase (HN) gene

An ORF for the HN gene was found extending from nts 7,563 to 9,302. This sequence has a 95% identity to the previously reported sequence for a HPIV4B isolate (GenBank AB006958), with an identity of 92% at the a.a. level, although the predicted protein of SKPIV4 is longer by 5 a.a at the carboxy terminal. Comparison with previously determined sequences of HPIV4A shows identities of 86%, and 84% at the a.a. level [6].

### 2.8. The Large (L) gene

**Figure 5.**
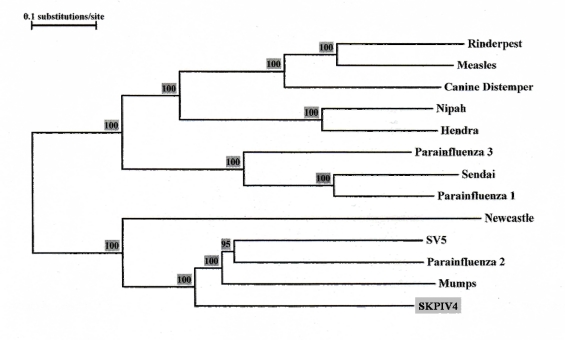
Phylogenetic tree built from an alignment of the Large (L) protein ORF sequences of several paramyxoviruses. The numbers at the node indicate the results of the Bootstrap analysis, expressed as percentages. The L ORF sequences were excerpted from the complete genome sequences listed in the legend of Figure 4.

The ORF coding for the large (L) protein of SKPIV4 spans nts 10025 to 16864, accounting for approximately 39% of the total genome. To date, this gene has not been sequenced for HPIV4. BLAST analyses of the nucleotide sequence failed to generate a significant match using the MEGABLAST program with standard parameters; the BLASTN program revealed several large segments with homology ranging from 66% to 70% with the L gene of mumps virus, and 65% to 68% with that of HPIV2. At the amino acid level, the predicted protein has 53% identity with the L protein of the mumps virus, with a BLAST score of 2,505; the second best match is with SV5 (52%; 2,431), followed by HPIV2 (51%; 2352). Figure 5 displays the phylogenetic tree calculated from an alignment of the L ORF sequence of SKPIV4 and the sequences of several paramyxoviruses.

### 2.9. The genomic termini

In order to determine the sequence of the termini, including the non coding regions, the genomic RNA was circularized using RNA ligase; the purified circular RNA was then subjected to RT-PCR using one primer anchored in the N coding region and the other in the L coding region. The RT-PCR yielded an amplicon of approximately 1.5 kb, containing the complete non coding terminal regions and the site of junction sealed by the RNA ligase. The amplicon was completely sequenced on both strands.

**Figure 6.**
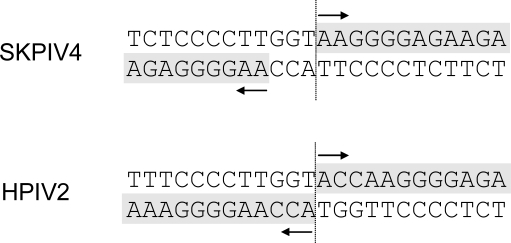
Top panel: hypothesized junction site of the extremities of SKPIV4 after RNA ligation and RT-PCR. Bottom panel: predicted junction site of the extremities of HPIV2 (NC_003443) after RNA ligation and RT-PCR.

To determine precisely the site of the junction between the ends of the genome, the sequence was examined for the type of extensive identity between the 3′ ends of the genome and antigenome exemplified by many paramyxoviruses [7], for example HPIV2 (Figure 6). This inspection led to a tentative identification of the junction site (Figure 6). To corroborate this hypothesis, a RT-PCR using primers Lend-1 and Lend-2 (Table 2) was done and, as predicted, yielded an amplicon of the expected length upstream of the junction. The final demonstration consisted in performing a 5′ RACE using primers 5RACE-1, 5RACE-2 and the primer supplied in the 5′ RACE kit. The sequence of the amplicon obtained by 5′ RACE showed the junction to be exactly as postulated in Figure 6.

## 3. Material and methods

### 3.1. Source of the HPIV4 strain

The virus was isolated from a nasopharyngeal swab submitted to the Clinical Virology laboratory of the Hospital for Sick Children (Toronto) for respiratory viruses detection.

### 3.2. Isolation and culture

The isolate was initially grown in primary rhesus monkey kidney cells as described [9]. The presence of the virus was demonstrated by hemadsorption with guinea pig erythrocytes. The virus was subsequently passaged in LLC-MK2 cells (American Type Culture Collection, Manassas, Virginia). Passage 5 stock was used for RNA extraction and sequencing.

### 3.3. Immunofluorescence microscopy

Detection of respiratory virus antigens was done by direct immunofluorescence microscopy. Briefly, cells were pelleted in a microfuge at 12,000 g for 3 min and resuspended in 100 μL of phosphate buffered saline (PBS). Five μL aliquots were spotted on a multi-well glass slide, air-dried and fixed with cold acetone. Wells were stained with different labeled monoclonal antibodies specific for influenza A, influenza B, parainfluenza 1,2,3 (Chemicon, Temecula, Ca). For the detection of parinfluenza 4, the parainfluenza 4 antibody FITC reagent (Parainfluenza 4: antibody FITC conjugate “Ready to Use” Reagent; Chemicon #5034) was used according to the manufacturer’s recommendations.

### 3.4. Electron microscopy

A cell suspension was obtained by scraping an infected cell monolayer with a sterile loop. The suspension was centrifuged for 2 min in a microfuge at 12,000 g, the supernatant discarded and the pellet resuspended in 1% ammonium acetate. Five μL of the suspension were applied to a Formvar and carbon coated electron microscopy grid and stained with 2% phosphotungstic acid, as described [10]. The grids were examined with a JEOL 1010 electron microscope at a magnification of 50,000 ×.

### 3.5. Extraction of viral RNA

Total RNA was extracted from aliquots of cell suspensions collected from culture infected with the parainfluenza 4 isolate (SKPIV4), using the TRIzol reagent (Invitrogen, Burlington, Ontario, Canada) as per the manufacturer’s recommendations. The RNA pellets were resuspended in ddH_2_O containing 10% of 100mM dithiotreitol (Invitrogen) and 5% of 20–40 U/μl RNasin (Promega, Mississauga, Ontario), and stored at −80° C.

### 3.6. Primer design for long RT-PCR

Primers used in the long RT-PCR were designed using Gene Runner v3.05 (Hasting Software), based on the sequences of HPIV4 (when available) and the sequences of other paramyxoviruses including HPIV1, HPIV2, HPIV3 and mumps virus.

### 3.7. Long RT-PCR

Long RT-PCR was done essentially as described [11, 12], with an elongation time optimized for each amplicon. Figure 3 illustrates the position of the overlapping amplicons that span the HPIV4 genome. Table 1 lists the sequence of the primer pairs used and the size of the corresponding amplicons.

### 3.8. RNA ligase mediated amplification of genome ends

The viral genomes, contained in the purified RNA extracted from cells infected with SKPIV4, were circularized by ligating the ends with T4 RNA ligase; the resulting circular RNAs were then purified. This was done using the GeneRacer kit (Invitrogen) as per the manufacturer’s instructions. The purified circularized viral RNA was then subjected to long RT-PCR, using the primers Para-GRacer-LS and Para-GRacer-NRS (Table 2). The resulting amplicon was then sequenced.

### 3.9. 5′ RACE

The 5′ RACE System procedure (Invitrogen) was used to determine sequence of the viral (genomic) RNA at the 5′ end, as per the manufacturer’s recommendations. Briefly, the single strand cDNA was synthesized using Superscript II reverse transcriptase and the primer 5RACE-1 (Table 2). The cDNA was then purified using the S.N.A.P. column followed by TdT tailing of the cDNA as per the manufacturer’s protocol. Five uL of the resulting dC-tailed cDNA was used as template in a PCR reaction consisting of 2 μL of primer 5RACE-2 (10 μM) and the supplied primer AAP, 5 μL of 10X PCR buffer, 5 μL of 25 mM MgCl_2_, 1 μL of deoxynucleoside triphosphate (200 μM), 0.5 μL of AmpliTaq Gold (Applied Biosystems Inc.), and 29.5 μL of molecular grade dd H_2_O. PCR was carried out in a Robocycler 40 thermal cycler (Stratagene) starting with one cycle consisting of denaturation at 94°C for 10 min, annealing at 53°C for 1 min and elongation at 72°C for 1 min 30 s, followed by 35 cycles at 94°C for 1 min, 53°C for 1 min, 72°C for 1 min 30s. The PCR product (375 bp) was submitted to sequencing.

### 3.10. Sequencing of amplicons

Amplicons from PCR reactions were subjected to electrophoresis on agarose gels containing the GelStar nucleic acid dye (Cambrex) and visualized on a Dark Reader transilluminator (Clare Chemical). Amplicons were sent to ACGT Corporation, (Toronto, Canada) for automated sequencing of both strands using *ad hoc* sequencing primers designed from previously obtained sequencing data. Initial reactions were done using the PCR primers.

### 3.11. Corroboration of the sequence

Additional PCRs and experiments were done to confirm the sequence of non-coding regions and of the L gene. Based on the complete sequence obtained, new primers were designed to amplify the noncoding regions between the ORFs and the amplicons were sequenced on both strands. The ORF coding for the L protein was completely re-sequenced with a different set of primers and overlapping amplicons. RT-PCRs targeting the non-coding genomic termini using primers at the very ends and primers within the N and L ORFs were done and the amplicons sequenced. The genomic RNA ligation was repeated using RNA from passage 4 infected cells. The 5′ RACE was repeated using RNA extracted from culture supernatant of passage 5 cells.

### 3.12. Sequence assembly and analysis

The individual sequence fragments were aligned and assembled using Gene Runner v. 3.05 (Hasting Software); editing was done using Gene Runner and Genedoc v 2.3 (distributed by Nicholas K.B. and Nicholas H.B.). Sequence alignments were calculated using ClustalX for Windows v.1.81 [13]. The GenBank database was interrogated using the BLASTN and BLAST search programs [14, 15]. Phylogenetic trees were inferred by using TREECON for Windows v.1.3b [16] using a distance method. The distance was calculated without corrections, taking gaps into account; the tree topology was inferred by the neighbor-joining method, and the trees were re-rooted at the internode. Bootstrap analyses were done with 1000 replicates.

## 4. Discussion

Among the known human paramyxoviruses, only the genome of HPIV4 had not been completely sequenced. The isolation of a HPIV4 from a clinical sample prompted the determination of the complete sequence, undertaken in this study.

The strategy used in the present study to assemble the full length HPIV4 genome, which involved overlapping large amplicons obtained by long RT-PCR and direct sequencing of the amplicons, provides some theoretical advantages over cloning in *E.coli* and sequencing clones, including obtaining directly the consensus sequence, and avoiding selection bias because of toxicity of some viral sequences to *E.coli* [17, 18].

The isolate used here was first identified as a paramyxovirus by electron microscopy, and as a HPIV4 by immunofluorescence microscopy. It was further typed as a HPIV4B by sequencing of the N gene and phylogenetic analysis (Figure 4); this was further confirmed by sequencing of the P/V, M, F and HN coding regions.

Among the subfamily *Paramyxovirinae*, the P/V region encodes for more than one protein (the number varies between virus species), in part through the mechanism of non-templated addition of G residues at the time of viral mRNA synthesis [1]. HPIV1 and HPIV3 encode the P protein through faithful mRNA transcription, and encode the V protein through non-templated insertion of G residues. In contrast, mumps virus and HPIV2 encode the V protein through faithful RNA transcription and the P protein through non-templated insertion. For HPIV4, Kondo *et al*. [3] performed direct mRNA cloning and sequencing and showed that HPIV4A and HPIV4B followed the strategy of the other rubulaviruses, encoding the P protein through non-templated insertion. Although viral mRNA purification from infected cells followed by cloning and sequencing was not carried out in this study, because of the identity of the sequences at the site where RNA editing would occur it is predicted that the isolate SKPIV4 sequenced here would behave in the same way.

The a.a. sequence for the HN protein of SKPIV4 is highly homologous to that reported by Bando *et al*., although it has five additional a.a. at the carboxy terminal. This is reminiscent of the finding of Sakaguchi et al [19], who reported that isolates of Newcastle disease virus (NDV) could be classified into three subgroups based on the different sizes of the HN protein caused by additional a.a. at the carboxy terminal and corresponding to three different viral lineages. This grouping correlated somewhat with virulence, although other determinants also play a role [19].

**Figure 7.**
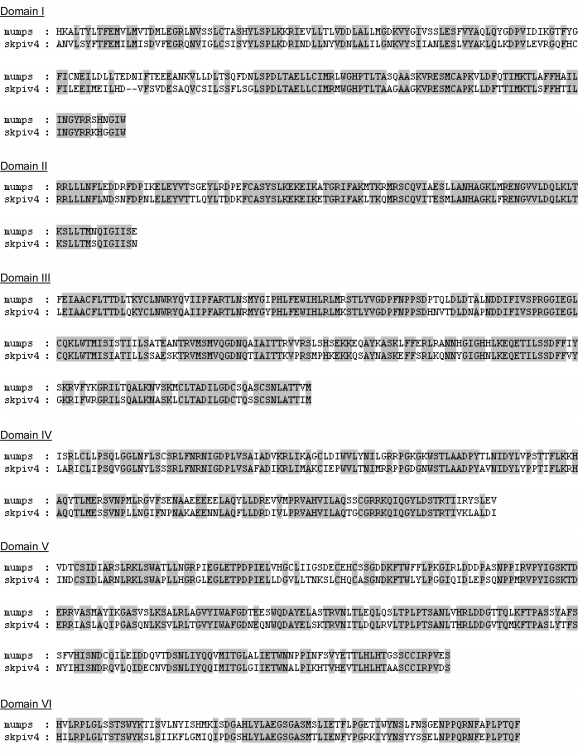
Alignment of the a.a. sequences of the L proteins of mumps virus and SKPIV4, within the 6 conserved domains of the L protein [8].

The L gene sequence presented here is the first ever determined for a HPIV4. The nucleotide sequence is unique, but shows significant homology with the L sequence of other rubulaviruses. The phylogenetic tree obtained from an alignment of the L gene sequences (Figure 5) displays essentially the same topology as the tree from the alignment of the N gene sequences (Figure 4). The L protein of paramyxoviruses is a very large protein with several enzymatic activities, including RNA directed RNA polymerase, 5′ end capping and methylation, and 3′ end poyladenylation. The L protein is involved not only in the synthesis of viral mRNAs but also in the synthesis of the antigenome and of the genome, these latter activities being dependent on the presence of soluble N proteins [1]. The L protein comprises 6 domains that are highly conserved among paramyxoviruses, and which are thought to contain the sites responsible for the enzymatic activities [8, 20, 21]; in particular, domain II has been proposed as a RNA binding domain, domain III as containing a conserved GDNQ motif involved in nucleotide polymerisation, and domain VI as involved in 5′ CAP formation [1]. Using the boundaries of the six domains in the L protein that were delineated for the mumps virus [8], it can be seen that within these domains there is very strong homology between the L protein of mumps virus and of SKPIV4 (Figure 7).

In this study the noncoding extremities of the HPIV4 genome were also sequenced, through ligation of the viral genome ends and RT-PCR. The junction sequence that was postulated by inspection of the sequence (Figure 6) and through comparison with the sequence of HPIV2 and the known complementarity of both ends of the genome was demonstrated to be correct through the use of the 5′ RACE procedure. A comparison with the ends of the genome of HPIV2 (or even of mumps) suggests that an additional ACC should be present at the 5′ end of the antigenome; further, such an addition would put the length of the complete genome at 17,364 nts, consistent with the “rule of six” [1]. It may be argued that since the sequence reported here is a consensus sequence determined by direct sequencing of amplicons, the complete, undamaged sequence could be present only in a minority of molecules and not be detected unless cloning and sequencing of many clones is performed. However, the addition of a ACC group would create a Kpn I restriction site at the junction (Figure 6); digestion of the amplicon with Kpn I failed to show even a partial digestion (data not shown), suggesting that if amplicons with the ACC group existed, they were indeed very rare and would require the sequencing of a very large number of clones to be detected.

The “rule of six” was initially formulated based on observations made on the Sendai virus [1]. Other studies using subgenomic replicons or defective interfering particles (DIs) of SV5, HPIV3 and Newcastle disease virus have shown that for subgenomic replicons, adherence to the rule of six was not essential, although polyhexameric length was associated with a greater replicative efficiency [22–24]. Despite the fact that most sequences of HPIV2 have a polyhexameric length, the reported sequence of the Toshiba strain (GenBank NC_003443) had a length of 15,646 nts; transfection of non polyhexameric cDNAs based on this strain yielded infectious HPIV2 virions [25] although the genomes of the progeny virions were not completely re-sequenced. A systematic investigation of this issue was undertaken by Skiadopoulos et al [26]; they found that non-polyhexameric full length cDNA clones reliably yielded infectious progeny viruses after transfection, but sequencing of the resulting genomes demonstrated the acquisition of compensatory mutations (insertions or deletions) that made the genomes compliant with the rule of six. Thus, even if the “rule of six” is not as stringent as initially formulated, it remains nonetheless a powerful constraint on the genomes of the subfamily *Paramyxovirinae*. It is possible that the isolate sequenced in this study would have lost some nts by passage 4 and 5; it is also possible that our experimental approach failed to capture some nts at the termini, possibly through damage prior to ligation. Based on sequence comparison with other rubulaviruses, it would seem likely that an additional “ACC” at the 5′ end of the antigenome would be present in the “complete” sequence of HPIV4B. The final elucidation of this point may have to await for reverse genetics experiments [26].

Another surprising characteristic of the sequence is its length; at 17,361 nts it is the largest known genome within *Rubulavirus*. A comparison of the ORFs of SKPIV4 with that of other rubulaviruses (Table 3) shows that overall SKPIV4 tends to have longer ORFs than other rubulaviruses, but not by very much.

A comparison with previously known sequences of HPIV4B and HPIV4A shows that the ORFs have identical lengths, except for that of HN, which encodes an additional five amino acids. However, comparing the length of the non-coding intervals between SKPIV4 and HPIV2 (Table 4) shows that most of the difference between the length of the two genomes is accounted for by non-coding sequences. Although non-coding sequences of HPIV4A and HPIV4B were never completely determined previously, sequencing of various genes (from either mRNAs or genomic RNAs) contained partial or complete intervening sequences [2–6] which allows for a lower bound estimate of the length of non coding intervals. As is readily seen from Table 4, these estimates are remarkably consistent with the findings from SKPIV4. Thus, most of the features that contribute to the length of the HPIV4 genome have in fact been observed previously.

Although HPIV4 has the longest genome within *Rubulavirus,* there are other viruses with larger genomes than HPIV2 or even HPIV4 within the subfamily *Paramyxovirinae*. For example, within the closely related genus *Avulavirus*, AMPV-6 has a genome of 16,236 nts (GenBank NC_003043); within the *Henipavirus* genus, Hendra and Nipah have genomes of 18,234 nts and 18,252 nts, respectively [27, 28]; two recently discovered paramyxoviruses still not ascribed to any genus, the J virus [29] and the Beilong virus [30], have even larger genomes of 18,954 nts and 19,212 nts, respectively.

In summary, with the likely exclusion of a small number of nts at one genomic end, this study presents the first complete genomic sequence from a single isolate of HPIV4B. In particular, it presents the first available L gene sequence for a HPIV4, and the first sequence available for several non-coding regions. These data fill an important gap in our knowledge of the human paramyxoviruses and should facilitate molecular investigation of this relatively less studied human respiratory virus.

## Figures and Tables

**Table 1.** Primers used in long RT-PCR to generate the amplicons illustrated in Figure 1; the lengths of the amplicons are also listed.

**PCR amplicon**	**Primer**	**Primer Sequence (5′→ 3′)**	**Size (bp)**
a	Para4-1	CGAACAATTTCTTCAAACAACTGAAGATCG	1,438
	Para4-2	CTGTTCATTCTGATAGTTGGAGTCTGGTGTG	
b	LongPara-P1	GGTTGCATTCAGGTTTCTCAATCGTTCAGGC	3,138
	LongPara-M2	GCCCCCATAGATCACTGATGCCTACGCTTAAC	
c	LongPara-M1	CCGATCCACACGAATGAGGGGTATACATCTAGAG	4,266
	LongPara-HN2	CGTAAGGAGTGACGAATGTGAGTGGGTAAGACGAAC	
d	LongPara-HN1	GCTCAGTGGTTGCTGTCCTTGACGGATGTTTAC	3,545
	LongPara-2	GGAGTGATTTCGTCAACTTAAGCTGATCAAGAACTACACCG	
e	LongPara-L1	GGAGATACCAAGCAATAATACCCTTTGCTAGAAC	3,013
	LongPara-L4	GCTGTTACATGGATAAGGATGTATATTTGGGTTTG	
f	LongPara-L5	TAGCTGTGCAATGTCTCATGTGGGGCGTTAAAACC	1,733
	LongPara-L6	GTCGTACAGTATCCCGGATTGAACTGCGTAAAACTCACC	

**Table 2.** Primes used in the sequence determination of the extremities of the viral genome. Primers Para-GRacerLS and Para-GRacer-NRS were used to synthesize by RT-PCR the amplicon containing the junction (see Figure 6).

**Primer**	**Primer Sequence (5′→ 3′)**
ParaGRacer-LS	CTGATAATCAAAAGATCCTACAAGCAGGTGG
ParaGRacer-NRS	CAGATGATGATACGGCAAGTCGGAGG
LEnd-1	CTTTAGAAATGAATGAGCAAGTAGTCG
LEnd-2	CAGATTTGTCTAGTGAGGATGTTGTC
GSP1-v.2	GAAAGATACGGAGACGAGACAAC
GSP2-v.2	CAACATCCTCACTAGACAAATCTG

Primers Lend-1 and Lend-2 were used in a RT-PCR predicted to yield an amplicon upstream of the junction; these data allowed the design of the primers used in the 5′ RACE; Primers 5RACE-1 and 5RACE-2 were used, along with the AAP primer (Invitrogen) in the 5′ RACE reaction.

**Table 3.** Comparison of ORF lengths between SKPIV4 and some rubulaviruses. For each ORF the length (including the stop codon) is given in nt; the length of the corresponding protein, in a.a., is given in parenthesis.

**ORF**	**SKPIV4**	**HPIV4B**	**HPIV4A**	**Mumps**	**HPIV2**
N	1656 (551)	1656 (551)	1656 (551)	1650 (549)	1629 (542)
P/V	1198	1198	1198	1174	1186
P^1^	(399)	(399)	(399)	(391)	(395)
V	(229)	(229)	(229)	225)	226)
M	1149 (382)	1149 (382)	1149 (382)	1128 (375)	1134 (377)
F	1632 (543)	1632 (543)	1632 (543)	1617 (538)	1656 (551)
HN	1740 (579)	1725 (574)	1722 (573)	1749 (582)	1716 (571)
L	6840 (2279)	N/A	N/A	6786 (2261)	6789 (2262)

Source of sequences; SKPIV4, GenBank EU627591; HPIV4A and HPIV4B, [2–6] and GenBank AB006958; Mumps virus, GenBank AF314558; HPIV2; GenBank NC_003443;

1 For the P protein, the addition of two non-templated G residues occurs at the stage of mRNA synthesis.

**Table 4.** Comparison of the lengths, in nt, of the non-coding intervals between the ORFs, for SKPIV4 and several rubulaviruses. Source of sequences: as in Table 3.

**Non-coding interval**	**SKPIV4**	**HPIV2**	**HPIV4B**	**HPIV4A**
5′ NC	154	156	≥ 33	≥ 33
N-P/V	285	207	≥ 284	284
P/V-M	295	300	294	294
M-F	494	176	≥ 483	≥ 483
F-HN	699	372	699	720
HN-L	722	260	≥ 527	≥ 529
3′ NC	497	65	N/A	N/A
